# The Effect of Static Cupping Therapy in Non-specific Low Back Pain for Primary Dysmenorrhea

**DOI:** 10.7759/cureus.29771

**Published:** 2022-09-30

**Authors:** Aleena S Siddiqui, Sabih N Khan, Nikita Narwade, Shrikant Mhase, Aniruddha Thorat, Wruchika Nagrale, Roshan Umate

**Affiliations:** 1 Department of Rehabilitation, Mahatma Gandhi Mission (MGM) School of Physiotherapy, Aurangabad, IND; 2 Department of Cardiorespiratory Physiotherapy, Mahatma Gandhi Mission (MGM) School of Physiotherapy, Aurangabad, IND; 3 Department of Community Physiotherapy, Mahatma Gandhi Mission (MGM) School of Physiotherapy, Aurangabad, IND; 4 Department of Physiotherapy, Mahatma Gandhi Mission (MGM) School of Physiotherapy, Aurangabad, IND; 5 Department of Research, Narendra Kumar Prasadrao (NKP) Salve Institute of Medical Sciences and Research Centre, Nagpur, IND; 6 Department of Research and Development, Jawaharlal Nehru Medical College, Datta Meghe Institute of Medical Sciences, Wardha, IND

**Keywords:** menstrual flow, prostaglandins, low back pain, static cupping therapy, primary dysmenorrhea

## Abstract

Dysmenorrhea is often referred to as painful menstruation with cramping sensations in the lower abdomen, resulting in discomfort. This pain commonly radiates to both the thighs and lumbosacral regions. A 23-year-old female presented with a complaint of severe abdominal pain during menses, accompanied by low back pain (LBP) that typically started at the onset of menstrual flow and lasted for the first 24-48 hours for which no analgesics were taken by the patient. The pain intensity was recorded on a numerical pain rating scale (NPRS) that showed 6 out of 10 for abdominal and low back pain during activity and 4 out of 10 at rest, on account of which a two-day supervised static cupping therapy protocol was devised for the patient. After treatment, the NPRS recorded a remarkable decrease in pain intensity to 2 out of 10 for abdominal and low back pain during activity, as well as at rest. As static cupping therapy is used to target deeper muscles in the lower back and abdomen; this case report aims to highlight the beneficial effects of static cupping on primary dysmenorrhea and related non-specific low back pain.

## Introduction

Dysmenorrhea, often known as painful menstruation, is characterized by intense cramping in the lower abdomen that is frequently accompanied by additional symptoms including sweating, headaches, nausea, vomiting, diarrhea, and shivering that happen shortly before or during the menses typically at the onset of menstrual flow, which lasts for the first 24-48 hours, and the prevalence of dysmenorrhea among females and adolescents in India ranged from 50% to 87.8% [1,2].

Dysmenorrhea is pelvic pain that begins immediately before or at the onset of menstrual flow and lasts for one to three days during a cycle. Prostaglandins (PGs), which act directly on the uterine muscles to raise the basal intrauterine pressure, as well as the strength and frequency of the myometrial contractions, are released at a higher rate as a result of the increased sensitivity of peripheral pain fibers [3]. Primary dysmenorrhea is characterized by painful menstruation that often starts in adolescence [4].

Low back pain (LBP) is one of the most widespread health issues globally and is frequently linked to primary dysmenorrhea [2]. LBP has a high prevalence worldwide, and as the population ages, more people will certainly become afflicted in the years to come. Chronic back pain causes physical, emotional, and socioeconomic changes and consequently high use of medicines and health resources for which cupping therapy is one of the recommended traditional Chinese medicine therapies for chronic pain reduction [5].

Static cupping uses glass or ceramic/acrylic cups on the affected areas, causing negative suction pressure to stimulate the skin. This suction pressure helps create a partial vacuum to form a heating effect within the cupping glass. This application technique, in turn, helps to improve local blood supply to the underlying tissues and lymphatic circulation, as well as relieve painful muscular tension to effectively treat the pain [6]. Therefore, this case report aimed to highlight the efficacy and degree of pain reduction in dysmenorrhea and related low back pain after a supervised cupping therapy regimen.

## Case presentation

A 23-year-old female presented with symptoms of primary dysmenorrhea, including significant lower abdominal pain that radiated to the lower back and caused discomfort. The patient reported that she had been experiencing painful menstruation every month, which typically started at the onset of menstrual flow and lasted for the first 24-48 hours. The pain intensity was recorded on a numerical pain rating scale (NPRS), which showed 6 out of 10 for abdominal and low back pain during activity and 4 out of 10 at rest; however, she did not undergo any clinical investigations, and no analgesics were taken by the patient for the same. The lower abdomen was the primary location of the sharp, intermittent pain, which also occasionally radiated to the lower back and inner thighs. The patient tried various home remedies such as the application of hot water bags over the abdomen and the lower back, which provided temporary relief for menstrual cramps. On observation, the patient presented with a stooped posture, exhibiting rounded shoulders and decreased curvature of the lower back. The patient experienced pain in the lower abdomen on palpation, and on assessment, the lumbar flexion range of motion was limited.

Therapeutic intervention

**Figure 1:**
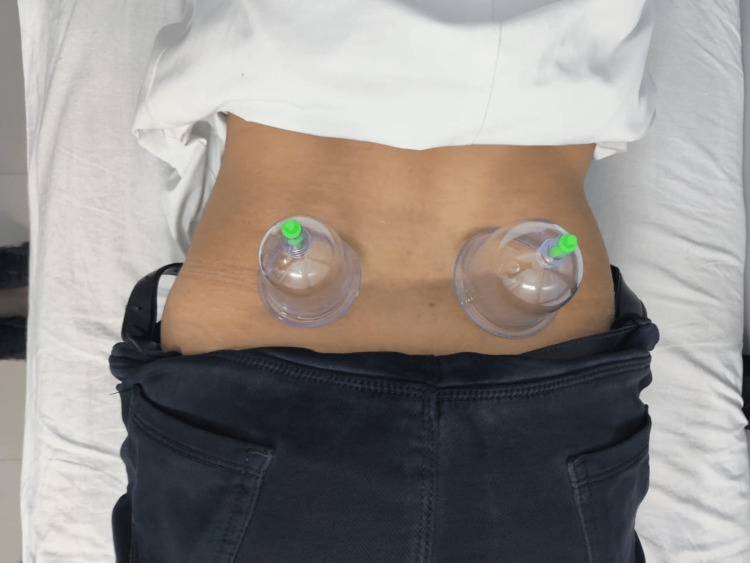
Two medium-sized cups applied on the lower back with the patient in the prone lying position.

**Figure 2:**
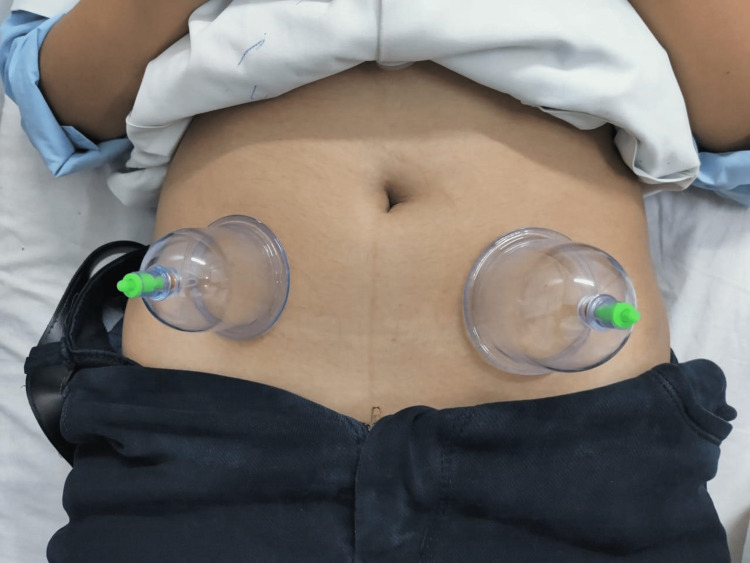
Two medium-sized cups applied below the umbilicus over the lower abdomen with the patient in supine position.

Before commencing static cupping intervention, the patient was briefed about the intervention protocol, and written informed consent was received. The patient was made aware that his medical records will be kept confidential. The size of the cups used depends on the area under consideration and the build of the patient; therefore, two medium-sized cups were applied exactly on the lower back with the patient in the prone lying position (Figure 1). Alternatively, with the patient in the supine position, two medium-sized cups were applied below the umbilicus over the lower abdomen (Figure 2) for approximately 10-12 minutes for each session. A medium suction strength of 300-500 mbar/three or four manual pumpings was used, and overall, five sessions were conducted [5].

As part of the five-session protocol, static cupping therapy showed a compelling response to low back pain and abdominal pain associated with dysmenorrhea. The pain subsided remarkably after static cupping therapy along with improved range of motion as shown in Table 1.

**Table 1:** Pre-treatment and post-treatment outcome measures. NPRS: numerical pain rating scale.

Outcomes	Pre-treatment values	Post-treatment values
Pain (lower back and abdomen)	6 and 4 on NPRS during activity and at rest, respectively	2 on the NPRS
Range of motion (lumbar region ) in centimeters (cm) using modified-modified Schober’s test		
Flexion	2	3
Extension	1	2
Left thoracolumbar rotation in degrees using goniometer	0-20	0-25

## Discussion

This case report focuses on analyzing the impact of static cupping therapy in non-specific low back pain for primary dysmenorrhea. When primary dysmenorrhea first appears, there is no obvious pelvic pathology to explain the low back and abdominal pain. But clinical studies have shown a physiological rationale for dysmenorrhea that is directly linked to elevated levels of endometrial and menstrual fluid prostaglandins (PGs). The first two days of menstruation are when PG levels are at their peak, causing significant abdominal pain and discomfort that extends to the lower back and thighs and interferes with everyday activities [7].

The treatment protocol should aim to relieve pain by affecting the physiological mechanism underlying menstrual pain, which is attributed to the release of PGs. Numerous studies show that due to potential adverse effects, pharmacological treatments should only be utilized at lower dosages for brief periods of time for treating non-specific low back pain. However, it has been hypothesized that non-pharmacological methods, such as physical activity, pain education, and manual treatment, function by increasing pelvic blood flow and catalyzing the release of beta-endorphins, which have non-specific analgesic properties [8].

The patient in this case report demonstrated remarkable recovery after the application of cupping therapy, which can be corroborated by several theories that have been proposed to support its benefits. Cupping therapy is believed to improve blood supply by hastening the metabolite removal and accelerating healing at the site of application [9]. Furthermore, it has been claimed that this method shifts the discomfort from one location to another, thereby eliminating pain. Numerous research has shown how cupping therapy has been frequently used in clinical settings to manage low back pain [5,6]. The improvement in the intensity of pain is also thought to be due to theories such as the pain gate mechanism and reflex zone.

## Conclusions

Although cupping therapy has been used for a long time owing to its efficacy and degree of reduction in pain intensity in dysmenorrhea, the validation and documentation of this treatment approach are extremely deficient. Therefore, it can be concluded from this case report that cupping therapy can be used in conjunction with other conventional interventions for treating abdominal and low back pain related to dysmenorrhea, as the former regimen is safe, cost-effective, and easily available.
